# Differential Expression of Iron Acquisition Genes by *Brucella melitensis* and *Brucella canis* during Macrophage Infection

**DOI:** 10.1371/journal.pone.0031747

**Published:** 2012-03-05

**Authors:** Linda Eskra, Jill Covert, Jeremy Glasner, Gary Splitter

**Affiliations:** 1 Department of Pathobiological Sciences, University of Wisconsin-Madison, Madison, Wisconsin, United States of America; 2 Department of Surgical and Radiological Sciences, University of California Davis, Davis, California, United States of America; 3 Biotechnology Center, University of Wisconsin-Madison, Madison, Wisconsin, United States of America; Universite de la Mediterranee, France

## Abstract

*Brucella* spp. cause chronic zoonotic disease often affecting individuals and animals in impoverished economic or public health conditions; however, these bacteria do not have obvious virulence factors. Restriction of iron availability to pathogens is an effective strategy of host defense. For brucellae, virulence depends on the ability to survive and replicate within the host cell where iron is an essential nutrient for the growth and survival of both mammalian and bacterial cells. Iron is a particularly scarce nutrient for bacteria with an intracellular lifestyle. *Brucella melitensis* and *Brucella canis* share ∼99% of their genomes but differ in intracellular lifestyles. To identify differences, gene transcription of these two pathogens was examined during infection of murine macrophages and compared to broth grown bacteria. Transcriptome analysis of *B. melitensis* and *B. canis* revealed differences of genes involved in iron transport. Gene transcription of the TonB, enterobactin, and ferric anguibactin transport systems was increased in *B. canis* but not *B. melitensis* during infection of macrophages. The data suggest differences in iron requirements that may contribute to differences observed in the lifestyles of these closely related pathogens. The initial importance of iron for *B. canis* but not for *B. melitensis* helps elucidate differing intracellular survival strategies for two closely related bacteria and provides insight for controlling these pathogens.

## Introduction

Iron is a required micronutrient for nearly all organisms as it is involved in a wide variety of essential metabolic processes. Although iron is abundant in the environment, it is not readily available inside host cells to prevent oxidative damage to itself or replication of pathogens. Pathogens, in particular, require efficient iron acquisition mechanisms to enable successful competition for iron in the highly iron-restricted environment of mammalian cells. When the intracellular iron concentration drops below a critical threshold, bacteria obtain iron by the direct uptake of heme or from iron-binding proteins by secreting small high affinity iron chelating compounds termed siderophores. A *Brucella* heme uptake mechanism and two *Brucella* siderophores (2,3-dihydroxybenzoic acid (2,3-DHBA) and brucebactin) have been identified [1], [2].

Since iron-siderophore complexes are too large to enter bacteria directly, bacteria have developed iron uptake systems. Iron uptake systems are typically composed of an outer membrane transporter, a periplasmic binding protein, and a cytoplasmic ATP-dependent transmembrane transport system [3]. In Gram-negative bacteria, the high affinity iron uptake complex, *ton*B/*exb*B/*exb*D, provides energy for the transport of iron complexes across the outer membrane to the periplasm [4]. Transport across the inner membrane requires a periplasmic binding protein and an ABC transporter [4].

Brucellosis is caused by Gram-negative *Brucella* spp. that replicate and survive within host monocytes, macrophages and dendritic cells. These intracellular bacteria possess few known virulence factors yet survive successfully within macrophages [5]. The species of *Brucella* are classified on the basis of host preference. Virulence is often associated with the lipopolysaccaharide (LPS) phenotype [6]. Smooth strains of *Brucella* express the O-chain of LPS, while rough species have substantially reduced or absent O-chain. The smooth strains, *B. melitensis*, *B. abortus*, and *B. suis* are pathogenic to humans. The natural rough strains, *B. canis* and *B. ovis*, are rarely pathogenic or non pathogenic to humans but are fully virulent for their natural hosts, dogs and sheep, respectively [7]. Despite close genome homology, *B. melitensis* and *B. canis* reportedly enter host cells via different routes [6], [8] and are found in different intracellular compartments [9]. The entry route of naturally rough *B. canis* is unknown [10]. The potential difference in entry of *B. melitensis* compared to *B. canis* would likely result in differing requirements needed by these two organisms for intracellular survival. Despite differences in virulence, *B. canis* and *B. melitensis* have in common approximately 3,520 genes based on genomes of 3,598 for *B. melitensis* and 3,523 for *B. canis* (www.PATRIC.org) suggesting that *B. melitensis* contains 78 unique genes, while *B. canis* has 3 unique genes.

We hypothesized that *B. melitensis* and *B. canis* might express different sets of genes for their intracellular survival, and we compared the expression profiles of genes of the smooth strain, *B. melitensis* that is a serious human pathogen, to the rough strain, *B. canis* that rarely causes human infections. Few studies have evaluated the expression of iron-related genes in *Brucella* spp., and even fewer studies have examined transcriptional changes in *B. canis*. Comparing transcript levels of these two *Brucella* species during infection of macrophages, we identified common and differentially expressed transcripts of iron acquisition genes. In contrast to most pathogens, *B. melitensis* did not increase its iron acquisition genes during early growth in mammalian cells. These data provide insight into the intracellular iron requirements of two *Brucella* spp. during macrophage infection.

## Materials and Methods

### Cell culture and infection

To compare the transcriptional changes occurring in brucellae grown in broth compared to macrophages, *Brucella canis* (*B. canis*) strain (ATTC #23365, strain RM6/66) and *Brucella melitensis* (*B. melitensis*) (ATCC#23456, strain 16 M) were grown to log phase on a shaker platform at 37°C in *Brucella* broth (Difco) for 1–2 days. Optical density (absorbance at 600 nm) was determined and an appropriate aliquot was added to brucella broth to achieve log phase within 24 h.

The mouse macrophage cell line, RAW 264.7 (ATCC TIB71), was maintained at 37°C with 5% CO_2_ in supplemented RPMI 1640 (10% bovine growth serum and 0.2 mM L-glutamine). One day prior to infection, 1–2×10^7^ RAW 264.7 cells were plated in T75 flasks. On the day of infection, RAW cells in a sample flask were counted, and the optical density of the bacteria was determined. Bacteria were added to the RAW cells to achieve an MOI of 100. After 90 min, extracellular bacteria were removed using three PBS washes followed by 30 µg/mL gentamicin (MP Biomedicals, Inc.) added to selected cultures. After 30 min, macrophages were washed three times and lysed with 0.1% Triton X-100 to determine bacterial CFUs at 2 h of infection. RPMI supplemented with 2 µg/mL gentamicin was added to cultures after 2 h and refreshed every 24 h. At 4, 8, 24, and 48 h cultures were washed and lysed as described at 2 h. Cell lysates were serially diluted and plated twice on brucella agar to determine bacterial CFUs. Experiments were repeated independently a minimum of three times. All work performed with *Brucella* spp. was conducted in a BSL3 environment.

### RNA extraction from broth grown bacterial cultures

Optical density (OD) measurements of bacterial cultures grown in brucella broth were obtained prior to RNA isolation. OD 600 readings of 0.5 to 0.8 were considered log phase. For RNA isolation, 4 ml of Protect (Qiagen) was added to 2 ml of log phase *B. canis* or *B. melitensis*. Cultures were incubated for 5 min at room temperature and then centrifuged at 1100×g for 10 min. Supernatant was decanted and the pellet resuspended in 200 µl of lysis buffer (TE containing 66 µg/ml Proteinase K (Epicentre) and 0.193 KU/ml ReadyLyse (Epicentre). Cells were incubated at room temperature for 10 min with vortexing every 2 min. RLT buffer (700 µl) with β-mercaptoethanol (Qiagen) was added and samples were transferred to 2 ml tubes containing 30–50 mg acid washed glass beads (1.0 mm). Bacteria were mechanically disrupted in a BeadBeater (Biospec Products, Inc) twice for 50 s at 4800 rpm. Tubes were centrifuged for 30 s at 13000 rpm to pellet beads and supernatant transferred to new tubes. RNA was isolated using RNeasy Mini Kit (Qiagen) according to the manufacturer's protocol. On-column DNase treatment was included in the isolation protocol. RNA for microarray experiments was obtained in duplicate from two separately conducted experiments. Yield and quality were determined using a spectrophotometer (Nanodrop Technologies) and Agilent bioanalyzer (Agilent).

### RNA extraction of intracellular bacterial from infected RAW 264.7 cells

RAW264.7 cells were infected at an MOI of 100 for 24 h. Flasks were washed twice with supplemented RPMI 1640 to remove extracellular bacteria and 2 ml of Protect diluted 2∶1 in PBS was added. Cells were transferred to centrifuge tubes, pelleted, and resuspended in 200 µl of lysis buffer. Cell lysis and mechanical disruption were performed as described above. RNA was isolated using RNeasy Mini Kit (Qiagen) with DNase treatment. Yield and quality were determined using a Nanodrop spectrophotometer and Agilent bioanalyzer, respectively.

### Enrichment of bacterial RNA

Bacterial RNA was enriched from RAW infected cells using MICROBEnrich kit (Ambion). Oligo Magbeads were washed with nuclease-free water and equilibrated in binding buffer. RNA was resuspended in binding buffer, mixed with capture oligo, incubated at 70°C for 10 min and annealed at 37°C for 1 h. The RNA/capture oligo mix was added to the magbeads and incubated at 37°C for 15 min. The oligo Magbeads were removed, and the supernatant was collected. Bacterial enriched RNA was assessed for yield and quality using a Nanodrop spectrophotometer and Agilent bioanalyzer.

### cDNA synthesis

Double-stranded cDNA was synthesized using Invitrogen Superscript II kit, substituting genome-directed primers (GDP) [11]for random hexamers. *Brucella* specific GDP (Table S1) were designed by Adel Talaat (University of Wisconsin-Madison). A 1.5 µl aliquot of GDP (1 µg/ul) were added to 10 µg RNA to a final volume of 11 µl and heated to 70°C for 10 min. A master mix containing 4 µl 5× first strand buffer, 2 µl 0.1 M DTT and 1 µl 10 mM dNTP mix was added to the sample. Following incubation for 2 min at 42°C, 2 µl of Superscript II was added and incubation continued at 42°C for an additional h. Second strand synthesis was performed by adding 91 µl nuclease-free water, 30 µl 5× second strand buffer, 3 µl dNTP mix, 1 µl 100 U/µl DNA ligase, 4 µl 100 U/µl DNA polymerase I and 1 µl 2 U/µl RNase H. Samples were incubated at 16°C for 2 h. T4DNA polymerase, 2 µl of 50 U/µl was added and incubated at 42°C for 5 min. The reaction was stopped by adding 10 µl 0.5 M EDTA. Following cDNA synthesis, a clean-up step of phenol∶chloroform∶isoamyl/alcohol was performed and DNA was precipitated using ammonium acetate. Yield and quality were determined using a Nanodrop spectrophotometer and Agilent bioanalyzer.

### Probe preparation and Hybridization

Samples determined acceptable for microarray hybridization were labeled and hybridized utilizing a Nimblegen protocol. Labeled samples (1.5 µg) were hybridized at 42°C to *B. melitensis* (Nimblegen A4357-001-01) or custom designed arrays (Nimblegen), using a 1× mixing chamber (Nimblegen) and Maui hybridization system (BioMicro). After 18–20 h, chambers were removed and slides were washed according to the manufacturer's protocol. Slides were scanned at 5 µm at a wavelength of 532 using a Genepix 4000B scanner (Molecular Devices Corporation, Sunnyvale, CA). Microarray experiments were performed in duplicate from two separately conducted experiments.

### Data Analysis

Fluorescent intensity levels were determined using Nimblescan (Nimblegen) software. The acquired images were normalized using quantile normalization and gene expression signals were generated using the Robust Multichip Average (RMA) algorithm. Genes were considered significantly changed if the fold change was ≥+/−2 and the Gamma Gamma (GG) model was ≥0.50 identified using EBArrays package in R [12]. Genome annotations were obtained from the RefSeq database at NCBI [13], [14]and the PathoSystems Resource Integration Center (PATRIC) [15]. All data is MIAME compliant and the raw data has been deposited in the MIAME compliant database Gene Expression Omnibus (GEO) platform database under accession number GSE34504, as detailed on the MGED Society website http://www.mged.org/Workgroups/MIAME/miame.html.

### Quantitative Real time PCR

Total RNA was isolated as described above. First strand synthesis was performed using 1 µg RNA, GDP and Superscript II reverse transcriptase, as described above. The resulting cDNA was diluted 1∶500 in DNase-, RNase-free water (Life Technologies, Rockville, MD). The single-stranded cDNA was then subjected to real time PCR using iQ SYBR green Supermix (Biorad) to quantify relative transcript levels. Sense and anti-sense primers for the tested genes are shown in Table S2. PCR was performed for 40 cycles at 95°C for 15 s, followed by 60°C for 30 s, with fluorescence detected during the extension phase. Control reactions were performed without RT to detect genomic DNA contamination. Levels of mRNA were quantified using the Pfaffl method [16]. Transcript levels of RAW 264.7 cell single-stranded cDNA were undetectable by real time PCR. Real time PCR experiments were performed in triplicate from two separately conducted experiments.

### Fluorescent staining

RAW 264.7 cells were plated one day prior to infection at 1×10^6^ macrophages/chamber in chambered coverglass slides. *B. melitensis* or *B. canis* were transformed with pBBR1MCS/GFP_uv_ containing the green fluorescent protein gene (*GFP_uv_*) under a constitutive Tac promoter with chloramphenicol resistance [17]. Transformed bacteria were grown overnight in brucella broth containing chloramphenicol, and GFP_uv_ fluorescence was similar between comparable numbers of bacteria (D. Magnani, personal communication). Macrophages were infected with *B. melitensis* or *B. canis* expressing GFP_uv_ in log phase growth with 100∶1 multiplicity of infection (MOI), as estimated by the optical density at 600 nm. The infection was allowed to continue for 6 or 24 h at 37°C with 5% CO_2_. At each time, macrophages were washed twice with PBS and fixed with 4% paraformaldehyde for 30 min at 4°C. Paraformaldehyde was removed and 100 µl of PBS with 1% BSA was added. Slides were examined by microscopy (Carl Zeiss, Halbergmoos, Germany). Macrophages containing fluorescent bacteria were digitally recorded using a charge-coupled device camera and Axiovert software.

## Results

### Infection of RAW macrophages

**Figure 1:**
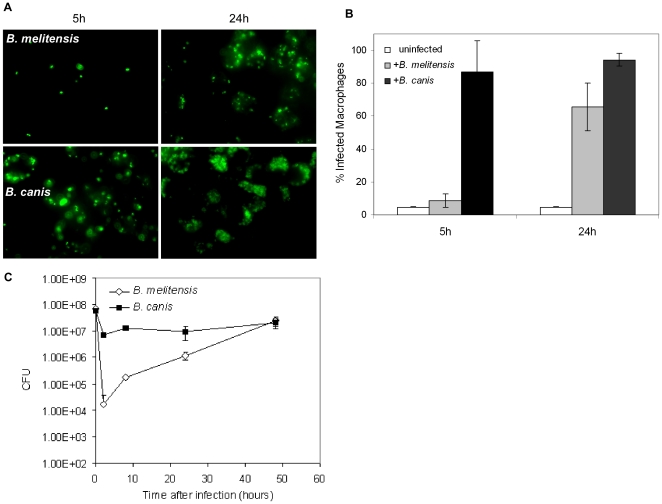
*B. melitensis* and *B. canis* infected macrophages. Panel A, fluorescent microscopy of *B. melitensis* and *B. canis* infected macrophages at 5 and 24 h. Panel B, flow cytometry analysis of the percent of macrophages infected with *B. melitensis* or *B. canis* at 5 and 24 h. Panel C, CFU determination of *B. melitensis* and *B. canis* from infected RAW cells at 4, 8, 24 and 48 h.

Internalization of rough versus smooth *Brucella* spp. differs in both uptake and trafficking patterns similar to observations of others [18], [19]. To determine the differences in bacterial uptake of *B. melitensis* and *B. canis*, RAW cells were infected for 5 or 24 h with *B. melitensis* or *B. canis* transformed with pBBR1MCS/GFP_uv_. Results in Figure 1A indicate that macrophages infected with *B. canis* at 5 and 24 h visually contained greater numbers of fluorescent bacteria than macrophages infected with *B. melitensis*. These observations were supported by flow cytometry of infected cells, Figure 1B indicating a greater number of macrophages were infected with *B. canis*. To determine the intracellular survival and replication of *B. melitensis* and *B. canis*, RAW cells were infected with bacteria and CFU were determined at 2, 8, 24 and 48 h (Figure 1C). As previously reported [20], a greater number of rough bacteria were taken up by the host cell. The number of rough bacteria remained at a constant level for 48 h. In contrast, few *B. melitensis* entered the macrophages but replicated over time and by 48 h the number of *B. melitensis* in RAW cells was similar to *B. canis*.

### RNA Isolation and quality assessment

Isolation of high quality RNA from intracellular *Brucella* can be technically challenging but critical to successful gene array profiling. Therefore, RNA and cDNA quality was stringently assessed using an Agilent Bioanalyzer. Gel-like images of *B. melitensis* and *B. canis* RNA and cDNA are shown in Figure S1A and S1B, respectively. RNA A260/A280 ratios ranged from 1.8 to 2.1. DNA contamination was not observed in any of the samples. These results demonstrate high quality bacterial RNA and cDNA recovery for use in microarray profiling. Although contaminating host cell RNA was variable following enrichment for bacterial RNA, less than 2% of gene expression was significantly different between duplicate experiments (data not shown). In addition, to determine the effect of RAW cell contamination, cDNA from RNA isolated from uninfected RAW cells was synthesized using GDP. Importantly, no host cell signal was detected on the microarray indicating the GDP procedure did not amplify host RNA.

### Microarray analysis

#### Changes in *B. canis* gene expression during macrophage infection at 5 h

**Figure 2:**
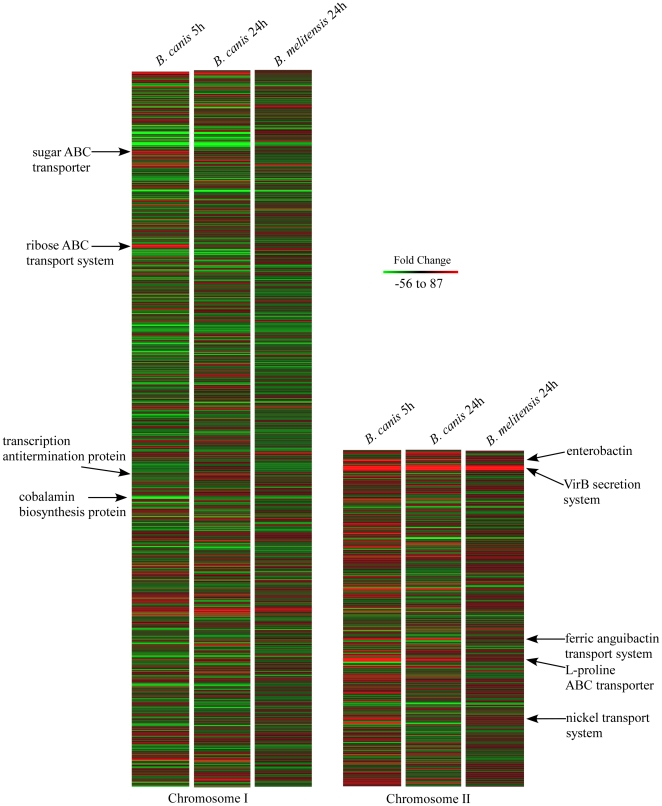
Heat map of *Brucella canis* (5 and 24 h) and *Brucella melitensis* (24 h). *Brucella* genomes from infected macrophages compared to respective broth grown bacteria. Selected genes are shown.

To identify common and differential transcriptional changes during macrophage infection, the gene expression profiles obtained from intracellular bacteria were compared to bacteria grown to log phase in brucella broth. Within the first few hours of infection, phagolysosome destruction of many ingested brucellae occurs and transcription of genes necessary for bacterial survival would be evident. By 24 h of infection, smooth brucellae containing vesicles have evaded bactericidal mechanisms and these vesicles transition to ER membranes for the long-term survival of brucellae. The trafficking pattern for *B. canis* is unknown. In the present study, we consider 5 h as early and 24 h as late in *Brucella* spp. infection of macrophages. Of the 2525 *B. canis* genes represented on the array, 630 (∼25%) showed statistically significant changes in expression (Figure 2 and Table S3) at 5 h of infection with a similar number of gene transcripts up (n = 305) and down (n = 325). Genes with the greatest increase (8–87 fold change) in transcription at 5 h of infection were the *virB* genes encoding the type 4 secretion system. Metal (Mg, Mn, Zn, Ni, Cu, and Fe) and numerous sugar-related transport genes, as well as genes associated with nucleic acid and DNA repair, and lipid synthesis also had increased transcripts. Additionally, at this early time was the increased transcription of genes associated with stress response such as the PhyR operon that encodes an alternative sigma factor of RNA polymerase which acts as a master regulator of general stress response [21], [22], [23], and genes associated with a response to nitric oxide. Genes whose expression decreased at 5 h of infection were generally associated with protein synthesis, oxidative metabolism, electron transport, and cell division. Changes in gene expression at 5 h suggest the attempt by *B. canis* to combat host defense mechanisms and minimize bacterial systems associated with metabolism and electron transport.

#### Changes in *B. canis* gene expression during macrophage infection at 24 h

To determine the gene expression changes in *B. canis* at later infection, the gene expression profiles from 24 h infection were compared to broth cultures. Of the 2525 *B. canis* genes represented on the array, 447 (∼18%) showed statistically significant changes in expression (Figure 2 and Table S4) at 24 h of infection with a similar number of genes increased (n = 199) and decreased (n = 248) in expression.

As the bacteria enter an intracellular long-term survival strategy, gene expression was compared between 5 h, early infection, and 24 h, later infection. Ten percent of the genes (267 genes) on the arrays showed significant changes in expression at 24 h compared to 5 h (122 increased and 14 decreased), Table S5. As expected the type 4 secretory system remained activated, as well as several metal transport systems, and the PhyR related operon [21], [23]. Transcript levels for nitrous oxide, nitrate and nitric oxide reductases, and sensory histidine kinase genes were decreased at 24 h compared to 5 h supporting the concept that the bacteria had survived initial phagolysosomal insult. Increased transcript levels were observed in flagellar-related genes, and two c-di-GMP related genes that are considered bacterial second messengers. A gene encoding dihydroxy-acid dehydratase (ilvD) was increased 4-fold that is also increased in expression in exponential Mycobacterial growth under acid stress [24]. Decreased transcript levels were observed at 5 h and 24 h in cyclic beta-1,2 glucan export ATP-binding protein, twin arginine transport system, Sec-dependent secretion system, electron transport systems, heat shock proteins, and chaperones. Similar decreases in the proteome expression for many of these systems were observed in *B. melitensis* by others [25]. However, transcripts decreased only at 24 h were catalase, glutaredoxins, and an operon (BCAN_A1142-1148) which is important for *Mycobacterium tuberculosis* to persist in a nutritionally limited macrophage by activating peroxynitrite reductase/peroxidase, allowing *M. tuberculosis* to resist host-reactive nitrogen intermediates [26]. The iron (III) dicitrate transport system (BMEII0535-0537/BCAN_B0764-0762 had fewer transcripts in *B. canis* infection at 5 h compared to 24 h (Table S5). Generally, fewer transport systems were activated at 24 h compared to 5 h.

#### Changes in *B. melitensis* gene expression during macrophage infection at 24 h

Of the 3108 *B. melitensis* genes represented on the array, 404 (13%) were significantly altered at 24 h post infection compared to broth culture (Figure 2 and Table S6). Of the 404 *B. melitensis* genes with altered expression, 113 had increased numbers of transcripts and 291 had decreased numbers of transcripts at 24 h. Similar to *B. canis*, transcript levels of *znu*A, sensory histidine kinases, and *mgt*A were increased, while cyclic beta-1,2 glucan export protein (*ndv*A), *fix*K, *dna*K, *gro*ES, *gro*EL, *uvr*A/B, electron transport associated genes were decreased. In contrast to *B. canis* infection, iron uptake related *B. melitensis* genes were decreased in infected compared to broth grown cultures. Transcript levels of *vir*B were also increased, with fold changes ranging from 3–10.

#### Comparison of differential gene expression between rough and smooth *Brucella* spp

The *B. canis* and *B. melitensis* arrays have in common 2358 genes, 1604 on chromosome I and 754 on chromosome II. To examine qualitative differences between the transcriptomes of rough and smooth bacteria, the expression profiles of *B. canis* and *B. melitensis* from infected macrophages were compared. A subset of genes that illustrate differences in expression patterns between rough and smooth *Brucella* is shown in Table 1. Expression of biotin related genes was decreased in *B. melitensis* but increased in *B. canis*. Biotin, a cofactor for fatty acid synthesis and linked to acetyl-CoA, is associated with bacterial growth and increases in amount with increased cell division [27] suggesting *B. canis* is metabolically active in macrophages at 24 h in contrast to *B. melitensis*. *ClpA* and *B* protease genes associated with unfolding proteins before degradation or unfolding proteins from aggregates to reach a function state, respectively [28], were decreased in expression in *B. melitensis* but not in *B. canis*. These Clp proteases require DnaK/DnaJ chaperones [29] and were also decreased in expression in *B. melitensis* and *B. canis* supporting the coordinated association of these genes as reported [28]. Clp protease in *B. suis* is not necessary for bacterial growth in macrophages is but is involved in temperature-dependent growth regulation, and in bacterial clearance from infected BALB/c mice [30]. Also, a number of other proteases including protease DO, also known as DegP or HtrA [31] that increases during stress [31], [32], [33], had decreased transcripts in *B. melitensis* and *B. canis* suggesting similar regulation of a number of genes between these two bacteria. Numerous electron transport genes and translation-related genes had decreased transcripts in both bacteria. The decrease in transcription of similar as well as dissimilar sets of genes indicates that unlike *B. canis*, *B. melitensis* silences many of its functions within the first 24 h of infection. Of particular interest, were genes involved in iron transport. Genes of the Ton B iron transport system, as well as the enterobactin synthesis system, had increased transcription in *B. canis* infection but were unchanged in *B. melitensis* suggesting differences in intracellular bacterial strategies or kinetics.

**Table 1:** Comparison of *B. canis* and *B.* melitensis genes altered in macrophages at 24 h.

BME Locus tag	Bov Locus tag	Product Name	Locus	Fold Change *B. melitensis*	Fold change *B. canis*
BMEI1935	BCAN_A0009	oligopeptide-binding protein APPA precusor	-	NC	4.31
BMEI1936	BCAN_A0008	oligopeptide transport system permease protein	*oppB*	NC	3.15
BMEI1937	BCAN_A0007	oligopeptide transport system permease protein	*oppC*	NC	2.98
BMEI1938	BCAN_A0006	oligopeptide transport -binding protein	*oppD*	NC	6.68
BMEII0489	BCAN_B0816	nickel transporter permease	*nikC*	NC	3.00
BMEII0491	BCAN_B0814	nickel transporter ATP-binding protein	*nikE*	NC	7.03
BMEII0493	BCAN_B0812	transcriptional regulatory protein, LYSR family	-	NC	2.25
BMEII0539	BCAN_B0760	hypothetical cytosolic protein	-	NC	2.37
BMEII0541	BCAN_B0757	sugar transport system permease protein	-	NC	2.57
BMEII0542	BCAN_B0755	sugar-binding protein	-	NC	5.25
BMEII0544	BCAN_B0753	SN-glycerol-3-phosphate transport ATP-binding protein	*ugpC*	NC	4.46
BMEII0548	BCAN_B0748	glycine betaine/L-proline transport ATP-binding protein	*proV*	NC	3.32
BMEII0929	BCAN_B0318	ribonucleotide-diphosphate reductase subunit beta	*nrdF*	NC	3.41
BMEII0930	BCAN_B0317	ribonucleotide-diphosphate reductase subunit alpha	-	NC	6.43
BMEII0931	BCAN_B0316	ribonucleotide reductase stimulatory protein	*nrdI*	NC	7.26
BMEII0932	BCAN_B0315	glutaredoxin	*nrdH*	NC	6.27
BMEII1009	BCAN_B0237	c-di-GMP phospodiesterase A	-	NC	2.13
BMEI0566	BCAN_A1477	soluble lytic murein transglycosylase	-	NC	3.43
BMEI1749	BCAN_A0204	glycerol-3-phosphate dehydrogenase	*glpD*	NC	−5.01
BMEI1753	BCAN_A0199	CYSQ protein	*cysQ*	NC	−2.98
BMEI1754	BCAN_A0198	bifunctional sulfate adenylyltransferase subunit 1	*cysNC*	NC	−21.33
BMEI1755	BCAN_A0197	sulfate adenylyltransferase subunit 2	*cysD*	NC	−36.36
BMEI1757	BCAN_A0196	aminotransferase	-	NC	−2.96
BMEI1758	BCAN_A0195	ttranscriptional activator, LuxR family	-	2.36	NC
BMEI1759	BCAN_A0193	B12-dependent methionine synthase	*metH*	NC	−2.17
BMEI1760	BCAN_A0192	hypothetical protein BMEI1760	-	NC	−2.17
BMEI1764	BCAN_A0188	oxidoreductase	-	NC	−56.25
BMEI1765	BCAN_A0187	phosphoadenosine phosphosulfate reductase	cysH	NC	−45.65
BMEI1766	BCAN_A0186	sulfite reductase ferredoxin)	cysI	NC	39.80
BMEI1767	BCAN_A0185	hypothetical protein BMEI1767	-	NC	−56.28
BMEI1768	BCAN_A0184	uroporphyrin-III c-methyltransferase	-	NC	−29.25
BMEI1379	BCAN_A0566	transcriptional regulator	betI	NC	−6.92
BMEI1380	BCAN_A0565	choline dehydrogenase	betA	2.07	−2.86
BMEII0906	BCAN_B0343	stress-response and acid-resistance protein	hdeA	3.72	−2.59
BMEI0070	BCAN_A2047	aquaporin Z	aqpZ	3.17	−2.11
BMEI0239	BCAN_A1848	Usg protein	-	3.10	−4.96
BMEI1841	BCAN_A0110	sulfate-binding protein precursor	-	2.51	−3.47
BMEI1746	BCAN_A0207	alcohol dehydrogenase	-	NC	−10.59
BMEI1840	BCAN_A0111	sulfate transport system permease protein CysT	cysT-1	NC	−3.82

#### Differential expression of genes involved in iron transport

The qualitative differences in iron acquisition genes between the transcriptomes of *B. canis* and *B. melitensis* isolated from broth versus RAW cells were compared. Forty-one putative iron response-related genes were selected for analysis (Table S7). Transcription of nineteen selected genes (>45%) were increased in *B. canis* but not in *B. melitensis* following infection of RAW cells comprising an “iron signature” for *B. canis* in macrophages indicating the transcription of a broad array of iron-related genes. Increased gene transcription included genes involved in the TonB and ferric anguibactin iron transport system, enterobactin production, iron uptake (phoPQ), and the FTR1 family of iron permease transport. The iron regulated outer membrane protein, *frpB*, was the only gene with increased transcript levels in infection for both *Brucella* species compared to broth cultured bacteria while the ferric uptake regulator protein gene, *irr*, was the only gene whose transcription was decreased in both *Brucella* spp. following infection compared to bacteria grown in broth cultures suggesting that transcription of at least 3 major iron acquisition systems are active in *B. canis* but not in *B. melitensis*.

### Validation of microarray data by Real Time PCR

To validate expression trends, microarray data were compared to data obtained by real time PCR. A representative group of genes was selected for confirmation with real time PCR. Levels of mRNA were quantified by the Pfaffl method [16] using 16 S rRNA as a reference gene. RT-PCR results were relatively consistent with the results obtained with the microarrays (Table 2), supporting that the microarray data reflects the changes in transcript levels observed during *Brucella* infections.

**Table 2:** Confirmation of microarray data using real time PCR.

Product Name	Gene ID	Species	FC(q PCR)	FC(microarray)
TonB protein	BMEI0363	B. melitensis	1.4	1.1
	BCAN_A1709	B. canis	4.2	5.1
exbD	BMEI0364	B. melitensis	0.8	1.2
	BCAN_A1708	B. canis	5.8	5.6
exbB	BMEI0365	B. melitensis	0.5	1.0
	BCAN_B1707	B. canis	2.3	2.9
ferric anguibactin transport	BMEII0606	B. melitensis	0.7	1.1
	BCAN_B0675	B. canis	4.0	4.3
ferric anguibactin-binding protein	BMEII0607	B. melitensis	1.3	1.3
	BCAN_B0674	B. canis	7.0	5.7
	BMEII0910	B. melitensis	7.1	11.1

## Discussion

*B. melitensis* and *B. canis* share extensive genomic similarity [34], [35]. While a genome comparison using PATRIC suggests a difference of ∼75 genes, we have previously reported that *B. canis* lacks only 38 genes present in *B. melitensis* with the majority of these missing genes found in genomic island 3 [7]. One distinctive genomic difference is the absence in *B. canis* of the homologous BMEI1435 gene encoding polysaccharide deacetylase contributing to a partially rough phenotype of *B. canis*. Beyond a few genomic differences, divergence in their virulence and phenotype likely arises from different genome usage to adapt to their environments. Few *B. melitensis* enter infected host cells, while many *B. canis* gain entrance to host cells which suggests entry by different routes [6], [8] and/or are in different intracellular compartments [9] requiring different intracellular survival strategies [18]. To gain further insight into potential differing intracellular bacterial strategies, gene expression profiles of *B. canis* and *B. melitensis* obtained from infected murine macrophages were compared to bacteria grown to log phase in brucella broth. Comparing the gene expression profiles of rough and smooth strains of *Brucella* identified potential differences in adaptation mechanisms used by these two *Brucella* species. For example, numerous iron acquisition operons were increased in expression in *B. canis* in macrophages compared to broth culture, while transcript levels for these systems were not increased in *B. melitensis* infection compared to broth culture.

### Acquisition of iron- the role of siderophores

The amount of iron available to an intracellular pathogen is extremely limited. In fact, one element of the host immune response during infection is the sequestration of iron. Consequently, intracellular bacteria have developed diverse mechanisms to assimilate sufficient amounts of iron, and these iron acquisition mechanisms are often closely linked to virulence [36]. When the intracellular iron concentration drops below a critical threshold, bacteria obtain iron from iron-binding proteins by secreting siderophores, which form complexes with Fe(III) and serve to ferry nearby iron to the bacteria [37]. *Brucella* spp. produce two siderophores: 2,3-DHBA [2] and brucebactin. The biosynthesis of 2,3-DHBA requires the *dhbC*, *dhbB*, and *dhbA* (homologs of *entC*, *entB* and *entA*) [3] contained within the 2,3 DHBA operon [38]. A functional *entE* homolog located between *dhbC* and *dhbB*, is involved in the biosynthesis of brucebactin in *B. abortus* [39] and a similar gene (BMEII0078 or BCAN_B0017) is present in *B. melitensis* or *B. canis*, respectively. *B. abortus* utilizes 2,3-DHBA to produce the more complex siderophore brucebactin [38]. Previous work [40] has shown that *B. abortus* grown in broth under iron limiting conditions produces 2,3-DHBA. While 2,3-DHBA production is not required for the survival and replication of *B. abortus* in cultured murine macrophages or virulence in mice [40], 2,3-DHBA is essential for virulence in pregnant cattle [38]. In *B. abortus*, DHBA is essential for bacterial growth on erythritol potentially explaining its role in abortion in cattle [41]. Surprisingly, the transcription of *dbhCEBA* was increased in *B. canis* infection but not significantly altered in *B. melitensis* infection. The increased transcription of iron transport in *B. canis* at 5 and 24 h suggests that early activation of these iron sequestering genes is an important strategy for *B. canis* intracellular survival. The production of 2,3-DHBA as well as brucebactin siderophores suggests an intracellular environment with limited iron availability in *B. canis* infected cells. In contrast, iron sequestration was not used by intracellular *B. melitensis* at 24 h and suggests that its intracellular strategy is to express few genes to maintain a clandestine survival strategy [5]. Regulation of iron related genes in *Brucella* spp. is poorly understood and have not been examined in *B. canis*. Future experiments are required to address the requirement of iron regulation and uptake differences among the *Brucella* species.

The roles of transcriptional regulators *irr* but not *rirA* has been examined in *Brucella* spp. [42]. The *irr* gene regulates brucebactin and 2,3-DHBA but is not required for *Brucella* virulence in mice [42]. In *Agrobacterium tumefaciens* these two regulators function under opposing iron conditions with Irr protein active under low iron conditions, inhibiting iron utilization and RirA protein active under high iron conditions, repressing iron uptake [43]. Although transcription of the *irr* gene was decreased in both *Brucella* spp., transcription of the *rirA* gene was unchanged suggesting that future studies are necessary to examine the potential roles of these transcriptional regulators in iron utilization and acquisition of *Brucella* spp.

### Iron transport

**Figure 3:**
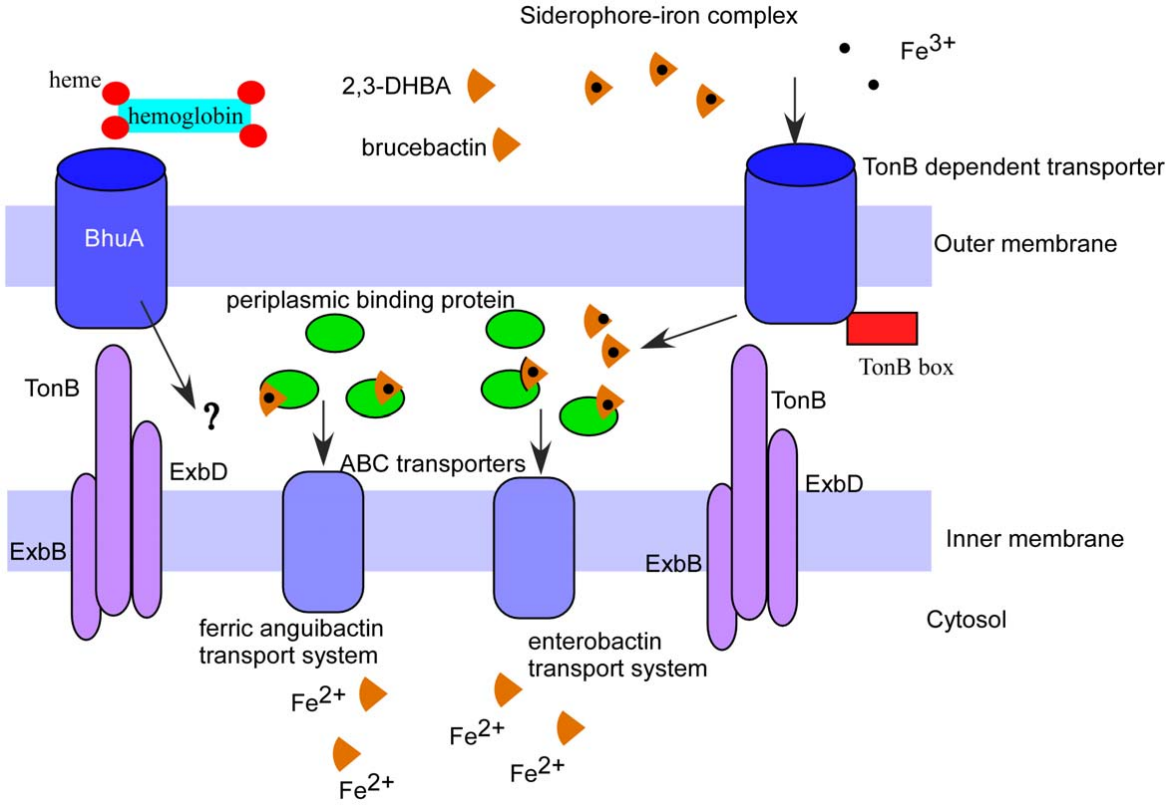
Model of iron transport in *Brucella* spp. Extracellular BhuA binds heme for transport across the outer bacterial membrane. The siderophores 2,3-DHBA and brucebactin bind iron(III) outside the bacteria and transport it through the outer membrane TonB dependent transporter. Function of the TonB dependent transporter requires TonB, ExbB and ExbD on the inner membrane. The siderophore-iron complex in the periplasmic space binds a periplasmic binding protein and is transported via one of several ABC transporters to the cytosol.

Once the siderophore-Fe(III) complex is formed, it is ready for uptake into the cell. Transport of iron across the outer and inner membrane of Gram-negative bacteria requires sophisticated iron uptake machinery, usually consisting of an outer membrane receptor, a periplasmic shuttling protein and an inner membrane transporter [4]. The bacterial outer membrane proteins, TonB-dependent transporters (TBDTs), bind and transport ferric siderophores across the outer membrane. Transport across the outer membrane requires a complex of three inner membrane proteins, TonB, ExbB and ExbD. TonB interacts with the TBDTs at the TonB box motif, Figure 3. Expression of TBDTs is highly regulated at both the transcriptional and post-transcriptional levels. The role of the TonB transport system has been well documented in *Salmonella entrica* [44] and *Shigella dysenteriae* [45] as *ton*B mutants in these bacteria exhibit reduced growth in host cells. *Shigella dysenteriae* mutants fail to grow in an iron rich intracellular environment suggesting the *ton*B complex may play a role in the growth and spread of intracellular bacteria in addition to iron acquisition [45]. *B. abortus* requires a TonB-dependent outer membrane protein for maintenance of chronic spleen infection in mice [1]. Additionally, the TonB complex is required for optimal uptake of the DHBA siderophore and/or citrate in *B. melitensis* [46]. As seen with the DHBA siderophore, transcription of *ton*B, *exb*B, *exb*D was increased in *B. canis* infection at 5 and 24 h. No significant difference in expression at the two time points was observed suggesting the long term need of this iron acquisition system. Transcription of *ton*B, *exb*B, *exb*D in *B. melitensis* infection was unchanged at 24 h compared to broth-grown bacterial cells supporting the greater covert lifestyle of *B. melitensis* compared to *B. canis*. Also, the TonB complex does not appear to be required for *B. melitensis* survival in macrophages or mice as deletion of these genes does not alter *B. melitensis* survival [46].

The absence for siderophores or the TonB complex requirement by *B. melitensis* suggests that this bacterium may utilize direct transport of heme, Fe(III), see below. The *frpB/bhuA* gene (BMEII0105) that encodes BhuA is involved in the utilization of heme as an iron source and required for *B. abortus* strain 2308 virulence in mice yet still requires the TonB complex for heme transport [1]. Deletion of Ton related genes in *B. canis* and the evaluation of *frpB/bhuA* in *B. melitensis* in future experiments would assess the potential mechanisms of iron acquisition for *B. canis* and *B. melitensis in vitro* and *in vivo* survival. Siderophores may play a role in placental colonization by *B. canis* in dogs, similar to the role of siderophores in *B. abortus* strain 2308 infection of pregnant cattle where siderophores are essential for virulence [38].

Transport of ferric siderophores across the inner membrane to the cytosol requires a periplasmic binding protein and an ABC transporter. Once the ferric siderophore enters the cytoplasm, ferric ion (Fe^3+^) is reduced to ferrous ion (Fe^2+^). The periplasmic binding proteins scavenge the Fe-siderophore, which interacts with the transmembrane permease unit and is transported across the inner the membrane to the cytoplasm. Three putative iron siderophore inner membrane transport systems have been identified in *Brucella* spp.: a metal chelator system (BMEI0657-0660 or BCAN_A1374-1371), an Fe(III) dicitrate system (BMEII0535-0537 or BCAN_B0764-762) and the Fe(III) anguibactin system (BMEII0604-0607 or BCAN_B0677-0674) [46]. The DstC protein (homolog to *fatC*) is encoded within BMEII0604-0607 and is required for optimal assimilation of DHBA and/or citrate [46]. DstC is a homolog of a permease iron (III) ABC transporter that, in Gram-negative bacteria, is involved in the uptake of iron through the inner membrane. Consistent with our previous observation, the components of the ferric anguibactin iron transport system (BCAN_B0677-0674) had increased transcription in *B. canis* infection at 5 and 24 h but the homologous genes were unchanged in *B. melitensis* infection. No transcriptional changes were seen with the metal chelate (BMEI0657-0660) and iron (III) dicitrate (BMEII0535-0537) transport systems at 24 h. The iron (III) dicitrate transport system transcripts were decreased at 5 h during *B. canis* infection.

In addition to the Ton complex and DstC, others have demonstrated that DugA proteins are required for optimal assimilation of DHBA and/or citrate [46]. DugA exhibits homology with the bacterial conserved GTPase YchF, and thus may have a role in iron assimilation in *Brucella*. *dugA* transcripts were also increased in *B. canis* but not *B. melitensis* infections. Thus, our results indicate that transcription of a large number of genes involved in siderophore synthesis and transport were increased in *B. canis* but not *B. melitensis* macrophage infection.

Although most bacteria rely on the ferric uptake regulator Fur, in some bacteria, like *Brucella* spp., regulation of siderophore production is mediated by an AraC-like transcriptional activator [47]. *B. abortus* AraC-like null mutant (*dhbR*) produced approximately 40% less siderophore compared to the wild type strain under iron-deprived conditions due to decreased transcription of *dhbCEBA* [48]. As with other bacteria [47], genes encoding an AraC-like transcriptional activator are repressed in iron-replete conditions. Consistent with our findings, the transcript level of the AraC-like transcriptional activator (BCAN_B1222) was increased in *B. canis* at 5 and 24 h but not in *B. melitensis* (BMEII0104) from infected cells.

### Alternative iron uptake mechanisms

In addition to the ferric transport described above, some Gram-negative bacteria also utilize alternative ferric iron transporters. In these systems, Fe3+ transport across the inner membrane is mediated by a ferric binding protein (Fbp) that serve as transport systems for iron delivered as transferrin and lactoferrin. The uptake of ferric iron does not require an iron (III) siderophore, outer membrane receptor protein, or TonB transport system. Previous studies have determined that the *Serratia marcescens* ferric uptake system, sfu, is composed of an ABC transporter SfuA, a periplasmic protein, SfuB, a cytoplasmic membrane protein, and SfuC, a membrane-bound protein. Upon entry into the periplasm, free iron is chelated by the iron-binding protein which initiates the transport of iron from the periplasm to the cytosol. Three potential sfu transport systems have been identified in *Brucella* spp.: BMEII0565-0567 (BCAN_B0726- 0724), BMEII0584-0586 (BCAN_B0702-0700) and BMEII1120-1123 (BCAN_B0119-0116) [46]. No differences in transcript levels for the three putative sfu transport systems of brucellae from infected cells compared to broth cultured bacteria were observed for the majority of these genes at 24 h. In concert with our transcriptional findings of *B. melitensis* iron related genes were proteomic findings of *B. abortus* that a broad number of iron capture and transport related proteins were reduced in expression early in infection suggesting these proteins did not play an active role early in bacterial pathogenesis but only later when the bacteria had begun replicating in the ER [49].

The failure to increase transcription of common iron transport systems in *B. melitensis* is highly unusual among intracellular bacteria. The overall regulatory mechanisms that control iron transport in *B. melitensis* in contrast to *B. canis* are unknown. Identifying bacterial signaling pathways that control iron transport in *B. melitensis* would provide greater understanding to the unique stealth survival strategy of this bacterium.

### Other transcriptional differences

Proteases can serve as virulence factors, and decreased transcription of numerous proteases was observed in *B. melitensis* and *B. canis*. Such changes might enable bacteria to silently persist in the face of aggressive antibiotic therapy [50]. The FeuPQ regulatory system, a major membrane sensory system was unchanged in *B. melitensis* but transcription increased in *B. canis*. FeuPQ is a regulator of greater than 100 genes in other bacteria [51]. One gene regulated by FeuPQ at 24 h was peptidoglycan transglycosylase (*mtgA*) that modifies the peptidoglycan structure. Again, these observations support the more silent entry into macrophages by *B. melitensi*s compared to *B. canis*. Catalase (*katA*), a protein that scavenges exogenous H_2_O_2_ was decreased in transcription at 24 h but was unchanged at 5 h in *B. canis* and was not altered in *B. melitensis* at 24 h. The observed decease or failure to increase in both *B. canis* and *B. melitensis* suggests this gene may not play a significant role in *Brucella* spp. intracellular survival, an environment where H_2_O_2_ would be anticipated. Our observations may explain a previous report that deletion of the homologous gene (BAB2_0848) in *B. abortus* did not alter the intracellular survival of *B. abortus* [52].

In summary, although *B. melitensis* and *B. canis* share much of their genomes, they function and survive quite differently in macrophages. Transcription of many genes was similar between these two organisms. However, major transcriptional differences were observed in genes involved in iron acquisition and transport. Genes of the TonB, enterobactin, and ferric anguibactin transport systems had increased transcription in *B. canis* but not *B. melitensis* during infection of macrophages. Iron is important for intracellular survival of bacteria. *B. canis* appears to require iron rapidly following entrance, while *B. melitensis* delays its requirement for iron for an extended period. In contrast to most pathogens, the delay in iron acquisition by *B. melitensis* appears as a unique strategy for early intracellular survival in mammalian cells. Transcriptional differences between *B. melitensis* and *B. canis* during macrophage infections underscore the value of analyzing several different *Brucella* species when probing *Brucella* pathogenesis.

## Supporting Information

Figure S1 **Gel-like images of RNA and cDNA of** ***B. melitensis*** **and** ***B. canis*** **.** Total RNA (23 S and 16 S bands) from *B. melitensis* (Panel A) or *B. canis* (Panel B) grown in broth is shown in lane (1). Eukaryotic (28 S and 18 S macrophage) and prokaryotic (23 S and 16 S) total RNA from infected RAW macrophages at 24 h *B. melitensis* in lane (3) or 5 h and 24 h *B. canis* in lane (3) and lane (6) post infection. Double stranded cDNA (lane 2, 5, and 8) was synthesized from *B. melitensis* or *B. canis* total RNA (lane 1) or bacterial enriched RNA (eRNA) (lane 4 or 7) from RAW macrophage infection, respectively.
(TIF)

Table S1 ***Brucella*** **Genome Directed Primers.**
(XLS)

Table S2 **Sequences of** ***Brucella*** **sense and anti-sense primers used in real time PCR.**
(DOC)

Table S3 **Gene expression profile of intracellular** ***B. canis*** **at 5 h compared to** ***B. canis*** **grown in broth.**
(XLSX)

Table S4 **Gene expression profile of intracellular** ***B. canis*** **at 24 h compared to** ***B. canis*** **grown in broth.**
(XLSX)

Table S5 **Gene expression profile of intracellular** ***B. canis*** **at 5 h compared to intracellular** ***B. canis*** **at 24 h.**
(XLSX)

Table S6 **Gene expression profile of intracellular** ***B. melitensis*** **at 24 h compared to** ***B. melitensis*** **grown in broth.**
(XLS)

Table S7 **Fold changes of putative iron acquisition genes of** ***B. melitensis*** **and** ***B. canis*** **from infected macrophages.**
(DOC)
