# Supplementary figures and images for: Differential Expression of Iron Acquisition Genes by Brucella melitensis and Brucella canis during Macrophage Infection
Source: PLoS One. 2012 Mar 5;7(3):e31747. doi: 10.1371/journal.pone.0031747 (PMC3293887; doi:10.1371/journal.pone.0031747)

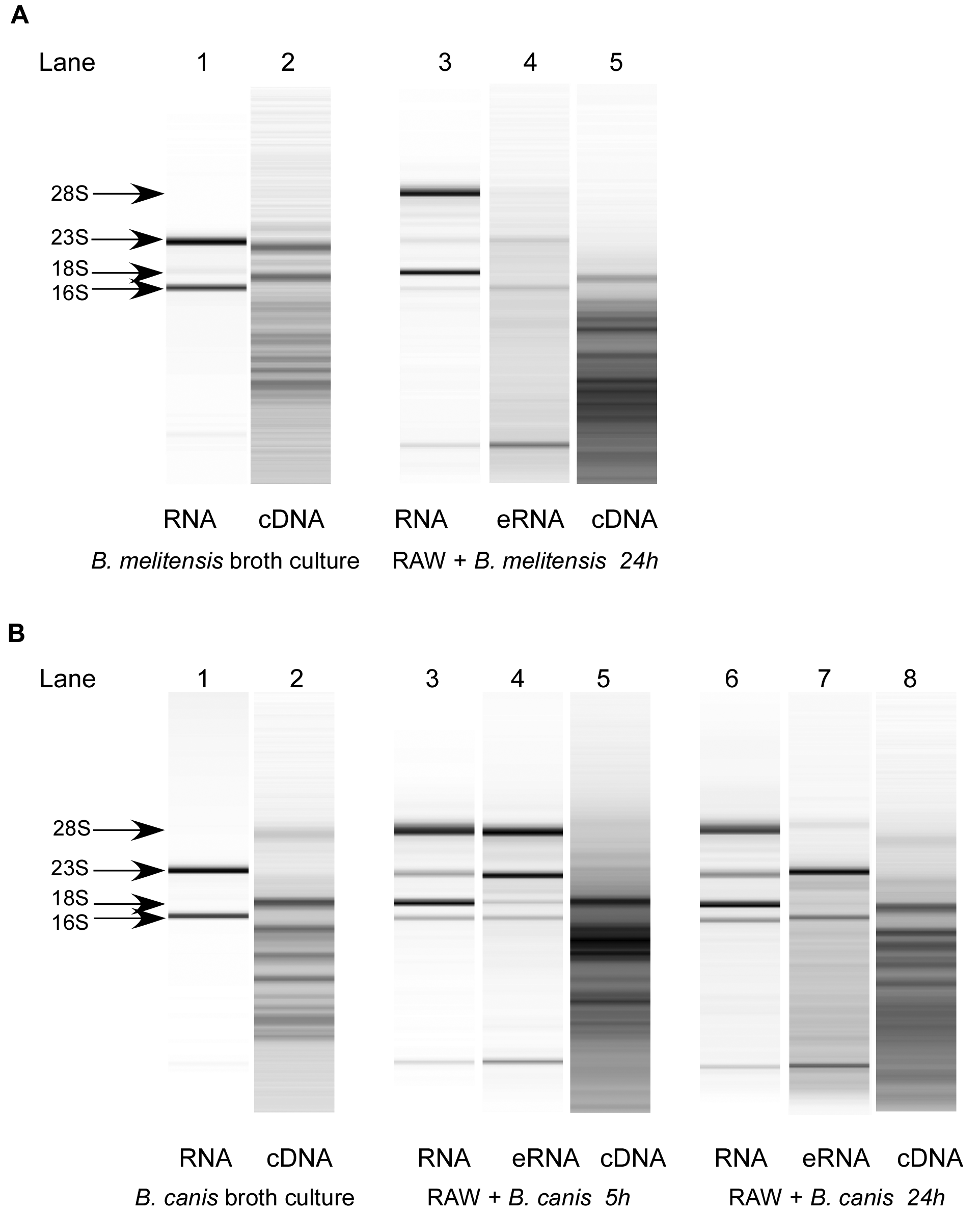

Supplement: Figure S1 — Gel-like images of RNA and cDNA of B. melitensis and B. canis . Total RNA (23 S and 16 S bands) from B. melitensis (Panel A) or B. canis (Panel B) grown in broth is shown in lane (1). Eukaryotic (28 S and 18 S macrophage) and prokaryotic (23 S and 16 S) total RNA from infected RAW macrophages at 24 h B. melitensis in lane (3) or 5 h and 24 h B. canis in lane (3) and lane (6) post infection. Double stranded cDNA (lane 2, 5, and 8) was synthesized from B. melitensis or B. canis total RNA (lane 1) or bacterial enriched RNA (eRNA) (lane 4 or 7) from RAW macrophage infection, respectively. (TIF) [file pone.0031747.s001.tif]
