# Supplementary material for: Differential Expression of Iron Acquisition Genes by Brucella melitensis and Brucella canis during Macrophage Infection
Source: PLoS One. 2012 Mar 5;7(3):e31747. doi: 10.1371/journal.pone.0031747 (PMC3293887; doi:10.1371/journal.pone.0031747)
Supplement: Table S2 — Sequences of Brucella sense and anti-sense primers used in real time PCR. (DOC) [file pone.0031747.s003.doc]

Supplemental Table S2. Sequences of sense and anti-sense primers used in real time PCR.

| BME | BCAN | Product Name | Primer |
| --- | --- | --- | --- |
| BMEI0363 | BCAN_A1709 | TonB | GAAGGGTCGATCACAAAGG |
|  |  |  | GTCCAGGCTGCAATCTCATA |
| BMEI0364 | BCAN_A1708 | exbB | GAGGGTCTCAAGGAACGTGT |
|  |  |  | CATGATACCCCAGACAGTGC |
| BMEI0365 | BCAN_A1707 | exbD | CTGGTGCTGCTCATCATCTT |
|  |  |  | CCTCCTTCAGCGTCACATAA |
| BMEII0605 | BCAN_B0676 | fatC | CAGACCGTCACCAATAATCG |
|  |  |  | TCAGGATATAGAGCGCATCG |
| BMEII0606 | BCAN_B0675 | fatD | ATAATCATGCCTGCAACAGC |
|  |  |  | TACGCTTGCCATTATTCTCG |
| BMEII0607 | BCAN_B0674 | fat binding protein | CAGAATGAATTCCGAGCTGA |
|  |  |  | ATGACAGCTTCGGTGTGAAG |
| BMEIr01 | BCAN_A1681 | 16S | GTGCTACAATGGTGGTGACA |
|  |  |  | GCGATTACTAGCGATTCCAA |
| BMEII0910 |  | glutamate decarboxylase | GCACTCTTCATTCGGGATTT |
|  |  |  | GGGTAATCTTCGGTGAAGGA |
