# Supplementary material for: Differential Expression of Iron Acquisition Genes by Brucella melitensis and Brucella canis during Macrophage Infection
Source: PLoS One. 2012 Mar 5;7(3):e31747. doi: 10.1371/journal.pone.0031747 (PMC3293887; doi:10.1371/journal.pone.0031747)
Supplement: Table S7 — Fold changes of putative iron acquisition genes of B. melitensis and B. canis from infected macrophages. (DOC) [file pone.0031747.s008.doc]

Table S7. Fold changes of putative iron acquisition genes of *B. melitensis* and *B. canis* from infected macrophages

| Product | Locus | *B. melitensis* (24h) | | *B. canis* (24h) | | *B. canis* (5h) |
| --- | --- | --- | --- | --- | --- | --- |
|  |  | Locus  Tag | Fold  Change | Locus  Tag | Fold  Change | Fold  Change |
| TonB protein | *tonB* | BMEI0363 | NS | BCAN_A1709 | 5.1 | 6.2 |
| exbD protein | *exbD* | BMEI0364 | NS | BCAN_A1708 | 5.4 | 4.5 |
| exbB protein | *exbB* | BMEI0365 | NS | BCAN_A1707 | 2.7 | 2.0 |
| enterobactin synthetase, component F | *entF* | BMEII0076 | NS | BCAN_B0019 | 2.4 | 2.5 |
| enterobactin synthase subunit E | *entE* | BMEII0078 | NS | BCAN_B0017 | 3.0 | NS |
| isochorismatase | *entB* | BMEII0079 | NS | BCAN_B0016 | 2.7 | NS |
| 2,3-dihydroxybenzoate-2,3-dehydrogenase | *entA* | BMEII0080 | NS | BCAN_B0015 | 3.9 | NS |
| iron(III) dicitrate transport ATP-binding protein | *fecE* | BMEII0604 | NS | BCAN_B0677 | NS | NS |
| ferric anguibactin transport system | *fatC* | BMEII0605 | NS | BCAN_B0676 | 5.3 | 4.5 |
| ferric anguibactin transport system | *fatD* | BMEII0606 | NS | BCAN_B0675 | 4.5 | 5.3 |
| ferric anguibactin transport system |  | BMEII0607 | NS | BCAN_B0674 | 5.7 | 3.5 |
| hypothetical protein |  | BMEII0882 | NS | BCAN_B0387 | 2.9 | 3.6 |
| iron permease, FTR1 family |  | BMEII0883 | NS | BCAN_B0386 | 3.6 | 3.7 |
| hypothetical protein |  | BMEII0884 | NS | BCAN_B0385 | 2.9 | 3.7 |
| sensor protein | *phoQ* | BMEI1336 | NS | BCAN_A0620 | 3.2 | 3.0 |
| transcriptional regulatory protein | *phoP* | BMEI1337 | NS | BCAN_A0619 | 2.3 | 2.7 |
| peptidyl-tRNA hydrolase | *pth* | BMEI0480 | NS | BCAN_A1573 | 2.3 | NS |
| 50S ribosomal protein L25/general stress protein | *dugA* | BMEI0481 | NS | BCAN_A1572 | NS | NS** |
| iron regulated outer membrane protein | *frpB* | BMEII0105 | 2.1 | BCAN_B1221 | 10.0 | 9.0 |
| outer membrane protein presursor | *nosA* | BMEII0297 | NS | Not on array |  |  |
| metal chelate outer membrane receptor |  | BMEI0657 | NS | BCAN_A1374 | NS | -3.7** |
| metal chelate periplasmic binding protein |  | BMEI0658 | NS | BCAN_A1373 | NS | NS |
| metal chelate transport system permease protein |  | BMEI0659 | NS | BCAN_A1372 | NS | NS |
| metal chelate transport ATP-binding protein |  | BMEI0660 | NS | BCAN_A1371 | NS | NS |
| iron(III) dicitrate-binding periplasmic protein |  | BMEII0535 | -2.00 | BCAN_B0764 | NS | -10.3** |
| iron(III) dicitrate transport system permease protein | *fecD* | BMEII0536 | NS | BCAN_B0763 | NS | -8.5** |
| iron(III) dicitrate transport system ATP-binding protein | *fecE* | BMEII0537 | NS | BCAN_B0762 | NS | -3.4** |
| iron(III)-transport ATP-binding protein | *sfuC* | BMEII0583 | -2.3 | BCAN_B0703 | NS | -2.4 |
| iron(III)-binding periplasmic protein precursor |  | BMEII0584 | -2.9 | BCAN_B0702 | NS | NS |
| iron(III)-transport system permease protein | *sfuB* | BMEII0585 | NS | BCAN_B0701 | 4.52 | 2.8 |
| iron(III)-binding periplasmic protein precursor |  | BMEII1120 | NS | BCAN_B0119 | NS | NS |
| iron(III)-transport system permease protein | *sfuB* | BMEII1121 | NS | BCAN_B0118 | NS | 2.1** |
| iron(III)-transport system permease protein | *sfuB* | BMEII1122 | NS | BCAN_B0117 | NS | NS |
| iron(III)-transport ATP-binding protein | *sfuC* | BMEII1123 | NS | BCAN_B0116 | NS | NS |
| iron(III)-binding periplasmic protein precursor |  | BMEII0565 | NS | Not on array |  |  |
| iron(III)-transport system permease protein | sfuB | BMEII0566 | NS | BCAN_B0725 | NS | 2.4 |
| iron(III)-transport ATP-binding protein | sfuC | BMEII0567 | NS | BCAN_B0724 | NS | NS |
| iron-responsive transcriptional regulator | rirA | BMEII0707 | NS | BCAN_B0562 | NS | NS |
| ferric uptake regulator protein | irr | BMEI1955 | -3.1 | BCAN_A2216 | -2.4 | -8.1** |
| ferric uptake regulator protein |  | BMEI0375 | NS | BCAN_A1696 | NS | NS |
| ferric uptake regulator protein |  | BMEI1563 | NS | BCAN_A0372 | NS | NS |

** significantly different from 24h infected macrophages
